# Short-term overfeeding of zebrafish with normal or high-fat diet as a model for the development of metabolically healthy versus unhealthy obesity

**DOI:** 10.1186/s12899-017-0031-x

**Published:** 2017-03-21

**Authors:** Kathrin Landgraf, Susanne Schuster, Andrej Meusel, Antje Garten, Thomas Riemer, Dorit Schleinitz, Wieland Kiess, Antje Körner

**Affiliations:** 1 Center for Pediatric Research Leipzig (CPL), University Hospital for Children & Adolescents, University of Leipzig, Liebigstraße 21, 04103 Leipzig, Germany; 2 Integrated Research and Treatment Center (IFB) Adiposity Diseases, University of Leipzig, Leipzig, Germany; 3 Institute of Medical Physics and Biophysics, University of Leipzig, Leipzig, Germany

**Keywords:** Obesity, Metabolic syndrome, Fatty liver, Hyperglycaemia, Adipose tissue, Adipocyte hypertrophy, Zebrafish, High fat diet

## Abstract

**Background:**

Obese individuals differ in their risk of developing metabolic and cardiovascular complications depending on fat distribution (subcutaneous versus visceral) and adipose tissue (AT) phenotype (hyperplasic versus hypertrophic). However, the exact mechanisms which determine whether an obese individual is metabolically healthy or unhealthy are not clear, and analyses of the underlying pathomechanisms are limited by the lack of suitable in vivo models in which metabolically healthy versus metabolically unhealthy AT accumulation can be specifically induced. In the current study, we aimed to establish a protocol for the use of zebrafish as a model for obesity-related metabolically healthy versus metabolically unhealthy AT accumulation.

**Methods:**

We overfed adult male zebrafish of the AB strain with normal fat diet (NFD) or high fat diet (HFD) for 8 weeks and compared parameters related to obesity, i.e. body weight, body mass index, condition index and body fat percentage, to control zebrafish fed under physiological conditions. In addition, we investigated the presence of early obesity-related metabolic alterations by quantifying blood glucose levels, plasma triglyceride and cholesterol levels, and by assessing ectopic lipid accumulation in the liver of zebrafish. Finally, we determined gene expression levels of marker genes related to lipid metabolism, inflammation and fibrosis in visceral AT and liver.

**Results:**

We show that 8-weeks overfeeding with either NFD or HFD leads to a significant increase in body weight and AT mass compared to controls. In contrast to NFD-overfed zebrafish, HFD-overfed zebrafish additionally present metabolic alterations, e.g. hyperglycemia and ectopic lipid accumulation in the liver, and a metabolically unhealthy AT phenotype with adipocyte hypertrophy especially in the visceral AT depot, which is accompanied by changes in the expression of marker genes for lipid metabolism, inflammation and fibrosis.

**Conclusions:**

In summary, we have established a method for the specific induction of metabolically distinct obesity phenotypes in zebrafish. Our results indicate that zebrafish represents an attractive model to study regulatory mechanisms involved in the determination of AT phenotype during development of metabolically healthy versus metabolically unhealthy obesity.

## Background

Obesity is characterized by an increase in adipose tissue (AT) accumulation in presence of positive energy balance and is a risk factor for metabolic and cardiovascular diseases, and premature mortality [1–3]. Depending on body fat distribution and AT phenotype obese individuals differ in their susceptibility to obesity-associated diseases, such as hepatic steatosis and type 2 diabetes [4–6]. Compared to the metabolically healthy obese (MHO) phenotype, which is characterized by a beneficial AT distribution and AT phenotype, the metabolically unhealthy obese (MUO) phenotype shows a deleterious AT distribution and phenotype with more visceral fat, bigger adipocyte size, and inflammatory processes [7]. However, the identification of mechanisms involved in the determination of body fat distribution and/or AT phenotype during obesity development are limited by the lack of suitable in vivo models, in which metabolically healthy versus metabolically unhealthy AT accumulation can be specifically induced.

Recently, the zebrafish (*Danio rerio*) has emerged as an alternative vertebrate model for the study of lipid metabolism and metabolic diseases, such as obesity, type 2 diabetes and hepatosteatosis [8, 9]. Organs and tissues of zebrafish are similar to those of humans in structure and function [10], and regulation of energy homeostasis at the neural and endocrine level is conserved [8, 11]. Moreover, diet-induced obesity in zebrafish shares common pathophysiological pathways with obesity in mammals [12–15]. Because of this, the zebrafish has become increasingly important for the identification of genes or potential drugs regulating lipid metabolism, AT accumulation and associated processes [9, 16].

We aimed to establish the zebrafish as a model for the analyses of mechanisms involved in the development of MUO or MHO, respectively. To this end, we evaluated different models of short-term diet-induced obesity by overfeeding of adult zebrafish with normal fat or high fat diet, respectively, and assessed the effect on AT accumulation, AT phenotype and the occurrence of associated metabolic alterations.

## Methods

### Zebrafish husbandry

Zebrafish were raised and maintained at 28 °C at a 14 h light : 10 h dark cycle. All animal experiments were performed according to European guidelines and approved by the local ethics committee (Landesdirektion Sachsen, Germany).

### Zebrafish feeding experiments

For feeding experiments, male zebrafish of the AB strain were used. We chose to use the AB strain for two reasons: First, the AB strain is widely used by the zebrafish research community and is frequently used for mutagenesis screens and gene knockdown approaches because embryonic lethals have been removed from the population allowing a more productive screen for embryonic phenotypes [17]. Second, among all strains available the AB strain has been most frequently used to study obesity and obesity-related processes in zebrafish [14, 15, 18, 19]. Male AB zebrafish were assigned to three dietary groups: One group was fed with peeled *Artemia salina* cysts (22% fat, 44% proteins, 16% carbohydrates; Aqua Schwarz) in a weight-maintaining amount (control; 5 mg artemia per fish per day), another group was overfed with artemia to induce an obese state under normal-fat-diet conditions (NFD-OF, 60 mg artemia) [14], and a third group was fed a combination of artemia (5 mg artemia) and egg yolk powder (59% fat, 32% proteins, 2% carbohydrates; Sigma; 30 mg) mimicking a high-fat-diet (HFD-OF) in an isocaloric amount compared to NFD-OF. Zebrafish were maintained at 10 fish per 3 L-tank and fed once per day. At week 8, zebrafish were fasted overnight and sacrificed. The feeding protocol used in this study was adapted from a previous study by Oka et al. showing that 5 mg artemia per day corresponds to the physiological energy requirement of an adult zebrafish [14]. Three independent feeding experiments were performed over a period of 18 months. Age of zebrafish included in each of the independent experiments was 92 days post fertilization (dpf), 99 dpf and 179 dpf, respectively, which corresponds to 3-6 months post fertilization (mpf).

Before the start (week 0) and at the end (week 8) of the feeding experiment, body weight and length of anaesthetized zebrafish was recorded, and body mass index (BMI) and Fulton’s condition index were calculated. Since we analysed 10 fish per tank individual tracking of fish during feeding was not possible and body weight, length and related parameters were averaged per tank. To exclude an effect of developmental and growth variations on our results, feeding groups were matched according to body length and body weight. Weight was determined every two weeks during the experiment without anaesthesia to preclude a bias of results by frequent anaesthesia. At week 8, body fat percentage was analysed using an EchoMRI 4in1 (EchoMRI^TM^). Fasting blood glucose was measured from the dorsal artery using a glucose meter (FreeStyle Freedom Lite, Abbott). For analyses of plasma triglyceride and cholesterol levels, blood samples were collected from the dorsal artery and pooled from all zebrafish per feeding group for one exemplary feeding experiment. Blood samples were centrifuged and plasma was collected from the supernatant. Triglyceride levels were determined using the LabAssay™ Triglyceride kit (Wako) and cholesterol levels were determined using the Amplex® Red cholesterol Assay Kit (Invitrogen) according to the manufacturer’s instruction.

### Magnetic resonance imaging (MRI)

MRI experiments were performed on a Bruker Advance DRX 300 MHz NMR spectrometer equipped with a 2.5 micro imaging unit (Bruker). Prior to the MRI measurements, the zebrafish were sacrificed, fixed in 2% (w/v) low melting agarose solution with 10% (w/w) sodium chloride. In order to determine the position of the fish, a TRIPILOT FLASH sequence was used for overview scans. A field of view of 16 x 16 x 250 mm was chosen to include the whole fish. The FOV was divided into 50 axial slices of 0.5 mm thickness. The plane resolution was set to 128 x 128 resulting in a voxel size of 7.81 mm^3^. Of each axial slice a fat and a water image were taken using a chemical excitation selective (CHESS) sequence. Water was excited by a frequency selective 300 Hz Gaussian pulse and fat by a 1 kHz EBURP pulse set on resonance respectively. In both cases a 4.6 kHz Sinc pulse was used for slice selective refocusing. Image processing was performed by an in-house written Python 2.7 script.

**Fig. 1:**
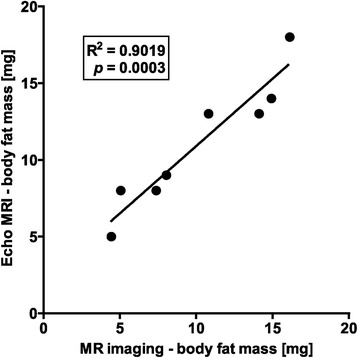
Comparison of methods for determination of body fat mass in adult zebrafish. Comparison of body fat mass (mg) determined by analyses of MR images (Bruker) and by EchoMRI 4in1 measurement (EchoMRI™) in 8 adult, male zebrafish of the AB strain at 6 months post fertilization showed a strong correlation between the two methods. Squared Pearson correlation coefficient R^2^ and *p-*value are shown in the scatter plot. NFD, normal fat diet; HFD, high-fat-diet; OF, overfeeding; MRI, Magnetic Resonance Imaging; AT, adipose tissue

In preliminary analyses, we compared body fat quantification based on MR imaging and Echo-MRI 4in1 measurement using 8 adult male AB zebrafish at 6 months post fertilization. For this, MR images were background corrected based on the noise distribution and the intensity was scaled by the corresponding receiver gain. Calculation of the fat-water ratio was performed for each pixel. Based on the fat-water ratio the fat volume per voxel was determined. Fat mass was calculated and compared to fat mass directly measured by Echo-MRI 4in1 (EchoMRI™). In fact, we detected a strong and significant correlation between the two approaches (Fig. 1).

### Histology

Whole Zebrafish were fixed in 4% paraformaldehyde, embedded in paraffin and cross-sectioned. Anatomically comparable sections of the subcutaneous and visceral region were stained with haematoxylin-eosin and microscopic images at 40x magnification were obtained. Image analyses were performed using ImageJ software (National Institutes of Health) [20–22]. In particular, for each fish subcutaneous and visceral adipocytes per field of view were counted and the cell area of each adipocyte was manually measured.

For preparation of cryosections from zebrafish liver, freshly isolated liver tissue was embedded in Tissue-Tek (Sakura Finetek), frozen at -80 °C, and cut using a cryostat (Thermo Scientific). Sections were dried, fixed in 4% paraformaldehyde, stained with Oil-Red-O, and analysed by microscopy.

### RNA isolation and mRNA expression analyses

Visceral AT and liver were homogenized in TRIzol reagent (Thermo Fisher Scientific) and total RNA was isolated from the aqueous phase using the RNeasy Mini Kit (Qiagen). 500 ng of RNA were reverse transcribed into cDNA using M-MLV Reverse Transcriptase (Invitrogen) and random hexamer primers (Promega). Quantitative *real-time* RT-PCR was performed as described [23]. Primer and probe sequences are listed in Table 1.

**Table 1:** Primer and probe sequences for quantitative *real-time* RT-PCR

Gene Symbol	Gene Full name	Method	Primer	Probe (5’-FAM, 3’-TAMRA)
*pparg*	*peroxisome proliferator-activated receptor gamma*	TAQMAN	F 5‘-GCTGCACAGGCGCTTCA R 5‘-CTCCAGCTCCTCCAGTTCCA	CAGAAAGCTTCACTCTCCGCTGATATGGTG
*fabp11a*	*fatty acid binding protein 11a*	TAQMAN	F 5‘-GGTTGACAAATTCGTAGGAACGT R 5‘-AACCCACACCTATAGCCTTCATG	AATGACCACCAGCGACAACTTTGACGA
*fasn*	*fatty acid synthase*	TAQMAN	F 5‘-ACACGGTTCACGCATTTGTG R 5‘-GACCCATCTTCCGTAGCATATCA	AGCTATTCAGGTTGCCCAGA
*srebf1*	*sterol regulatory element binding transcription factor 1*	TAQMAN	Predesigned (Dr03093012_m1; Thermo Fisher)	
*atgl*	*adipose triglyceride lipase*	SYBR Green	F 5‘-GCGTGACGGATGGAGAAA R 5‘-AGGCCACAGTAAACAGGAATAT	
*hsl*	*hormone-sensitive lipase*	SYBR Green	F 5‘-CGGCAAGGACAGGACAGT R 5‘-GCATGGAGAAAGAGGAGCT	
*lpl*	*lipoprotein lipase*	TAQMAN	F 5‘-CTGAGGGCTCTCGTTCATAAAGA R 5‘-AATCCATCAAAGACTGTAACTTCAATACA	CTCTCAAACATACCCGTGACCGTCCATC
*il1b*	*interleukin 1 beta*	TAQMAN	F 5‘- TCATCGCCCTGAACAGAATG R 5‘-TCACTTCACGCTCTTGGATGAC	AGCACATCAAACCCCAATCCACAGAGTT
*tnfa*	*tumor necrosis factor alpha*	TAQMAN	Predesigned (Dr03126848_g1; Thermo Fisher)	
*col1a1a*	*collagen, type I, alpha 1a*	SYBR Green	F 5‘-GCTTTTGGCAAGAGGACAAG R 5‘-TGTCTTCGCAGATCACTTCG	
*bactin2*	*beta-actin 2*	TAQMAN	F 5‘-TCCCCTTGTTCACAATAACCTACTAA R 5‘-CATACCGGAGCCGTTGTCA	AGCGATTTCCTCATCCATGGCTGTGT
*tbp*	*TATA-box binding protein*	TAQMAN	F 5‘-CCTGCGAATTATCGTTTACGTCTT R 5‘-ACGGCATCATAGGACTGAAAATG	TTGCTTCATAACCTGTCAGCATGGAGCA

### Protein isolation and immunoblot analyses

Proteins were isolated from the organic phase of the TRIzol (Thermo Fisher Scientific) tissue lysate, which was a side product from the RNA isolation procedure. Proteins were precipitated according to the manufacturer’s instructions. Equivalent amount of proteins were resolved by 10% SDS-PAGE and immunoblotting using antibodies directed against P-Akt (Thr308 for human Akt corresponding to Thr307 for zebrafish Akt, 244 F9; cat. no. 4056, Cell Signaling), total Akt (cat. no. 9272, Cell Signaling) and beta-Actin (cat. no. ab8227, abcam). Protein levels of P-Akt and total Akt were analyzed using ImageJ software (National Institutes of Health) and the ratio was calculated.

### Statistical analyses

Each feeding experiment was independently repeated two times. Statistical analyses were performed using GraphPad Prism 5 (GraphPad Software). Statistical tests used for each analysis are indicated in the figure legends.

## Results

**Fig. 2:**
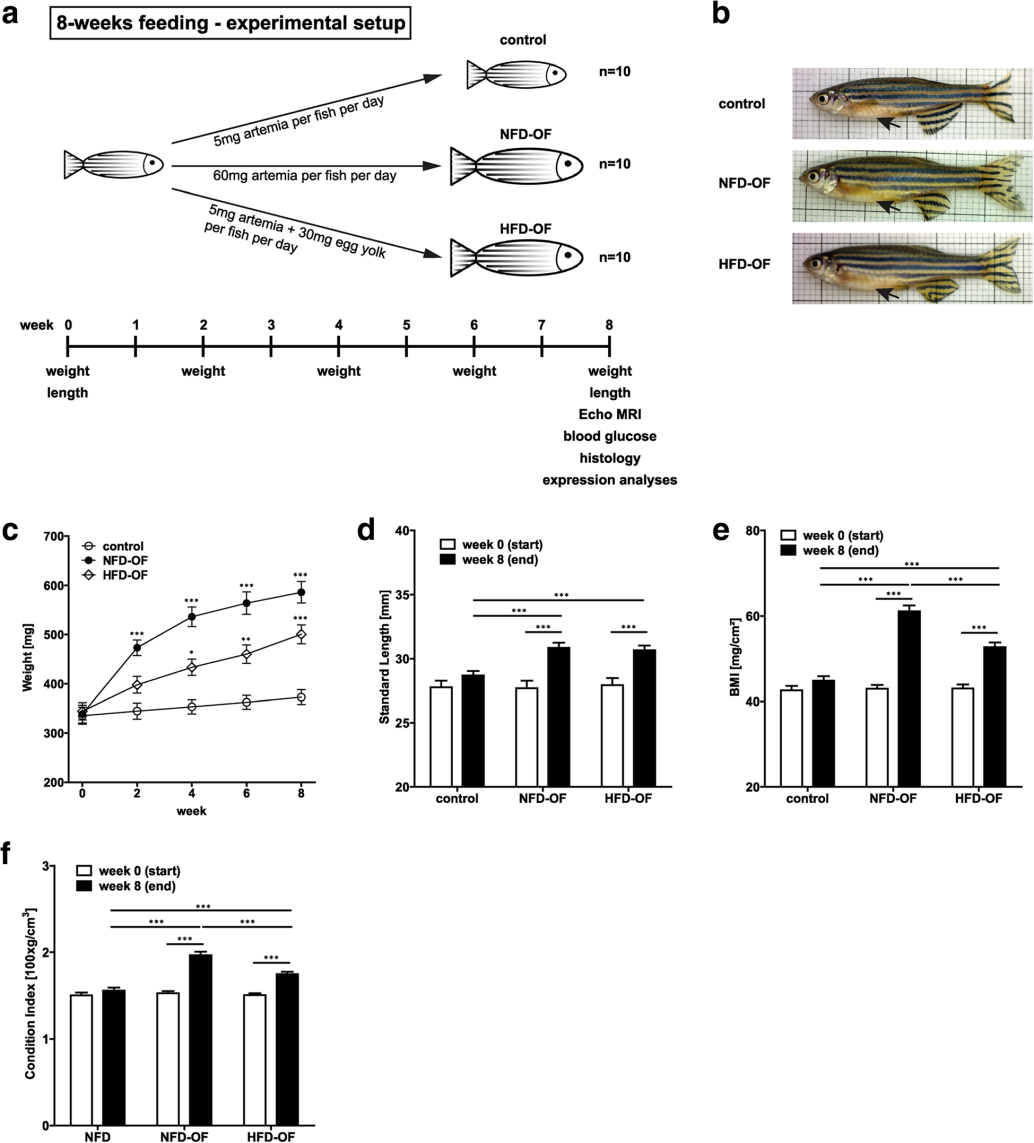
Overfeeding of adult zebrafish with either NFD or HFD leads to an obese phenotype. **a** Schematic overview of the 8-week feeding protocol and phenotyping of zebrafish. **b** Exemplary images of zebrafish included in each of the analyzed dietary groups at week 8 of feeding. Black arrows point to the abdominal region. Overfeeding of zebrafish with either NFD or HFD resulted in a significant increase in weight (**c**), standard length (**d**), BMI (**e**), and Fulton’s condition index (**f**) compared to control zebrafish. Statistical analyses were performed using Two-Way ANOVA and Bonferroni post-test and significant *p*-values are indicated. *, *p* < 0.05; **, *p* < 0.01; ***, *p* < 0.001; NFD, normal fat diet; HFD, high-fat-diet; OF, overfeeding; BMI, body mass index; MRI, Magnetic Resonance Imaging; AT, adipose tissue

**Fig. 3:**
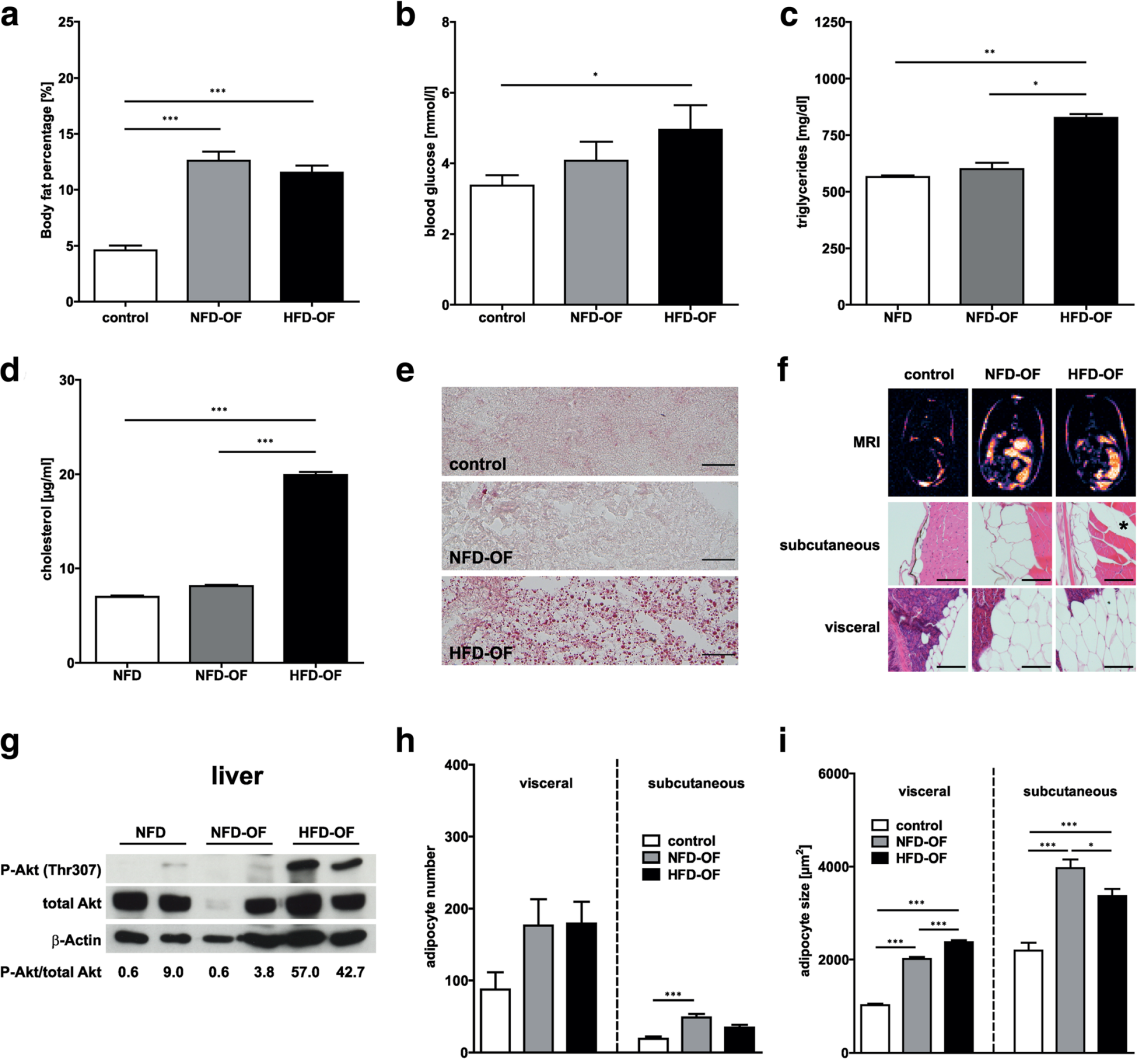
Phenotypic characterization of diet-induced obesity in zebrafish. Overfeeding of zebrafish with either NFD or HFD resulted in a significant increase in body fat percentage (**a**) compared to control zebrafish. HFD-OF but not NFD-OF zebrafish showed significantly elevated fasting blood glucose levels (**b**), triglyceride levels (**c**) and cholesterol levels (**d**), and a prominent accumulation of lipids in the liver as indicated by Oilred-O staining of liver sections (**e**). Fat distribution was analyzed by MRI and hematoxylin-eosin staining of zebrafish cross sections and revealed an increase in the amount of both subcutaneous and visceral AT in NFD-OF and HFD-OF compared to control zebrafish (**f**). Exemplary images of zebrafish included in each of the analyzed dietary groups at week 8 of feeding are shown. Asterisk indicates intramuscular fat deposition in HFD-OF fish. Immunoblot analyses of liver lysates showed increased phosphorylation of Akt at Thr307 (Thr308 in human) in HFD-OF compared to NFD-OF and control zebrafish. The ratio of P-Akt to total Akt was analyzed using ImageJ software and is given underneath the immunoblot images (**g**). Detection of β-Actin served as loading control. Visceral and subcutaneous adipocyte number and adipocyte size were determined from hematoxylin-eosin-stained zebrafish sections using ImageJ software and were increased after overfeeding with NFD or HFD (**h**, **i**). Statistical analyses were performed using One-Way ANOVA and Bonferroni post-test and significant *p*-values are indicated. *, *p* < 0.05; **, *p* < 0.01; ***, *p* < 0.001; Scale bar in (**h**) represents 50 μm, Scale bar in (**i**) represents 100 μm; NFD, normal fat diet; HFD, high-fat-diet; OF, overfeeding; BMI, body mass index; MRI, Magnetic Resonance Imaging; AT, adipose tissue

We assessed diet-induced obesity in zebrafish by overfeeding adult male zebrafish with normal-fat-diet (NFD-OF) or high-fat-diet (HFD-OF) compared to physiological control diet for 8 weeks (Fig. 2a). Both, NFD-OF and HFD-OF fish had an enlarged belly compared to control fish (Fig. 2b). Control zebrafish maintained their weight during the 8-week feeding experiment. While fish in the NFD-OF group almost doubled their weight, the weight gain of HFD-OF fish was not as prominent (Fig. 2c). The weight gain observed under overfeeding conditions was accompanied by an increased growth in both groups (Fig. 2d). Both NFD-OF and HFD-OF fish showed a significant increase in body mass index (BMI, Fig. 2e) and Fulton’s condition index (Fig. 2f), which was considerably higher in NFD-OF compared to HFD-OF fish. Body fat percentage was increased in both NFD-OF and HFD-OF fish but did not significantly differ between the OF groups (Fig. 3a). HFD-OF but not NFD-OF fish showed significantly elevated blood glucose levels compared to control zebrafish (Fig. 3b) and presented significantly elevated plasma triglyceride (Fig. 3c) and cholesterol (Fig. 3d) levels as well as a prominent ectopic accumulation of lipids in liver (Fig. 3e) and muscle (Fig. 3f). In line with this, immunoblot analysis of phospho-Akt and total Akt protein levels in liver lysates demonstrated higher levels of phospho-Akt in HFD-OF zebrafish compared to NFD-OF or control zebrafish indicating an early state of insulin resistance (Fig. 3g). Analyses of fat distribution by MRI and haematoxylin-eosin staining of zebrafish cross sections revealed an increase in both subcutaneous and visceral AT in NFD-OF and HFD-OF compared to control zebrafish (Fig. 3f). Both, NFD-OF and HFD-OF groups showed a tendency towards an increase in adipocyte number in visceral AT. In subcutaneous AT adipocyte number was significantly increased in NFD-OF fish, while there was only a marginal increase in adipocyte number in HFD-OF fish (Fig. 3h). Visceral and subcutaneous adipocytes were significantly larger in NFD-OF and HFD-OF compared to control fish. Importantly, HFD-OF zebrafish had larger visceral but smaller subcutaneous adipocytes compared to NFD-OF zebrafish indicating the presence of a metabolically unhealthy AT phenotype (Fig. 3i).

**Fig. 4:**
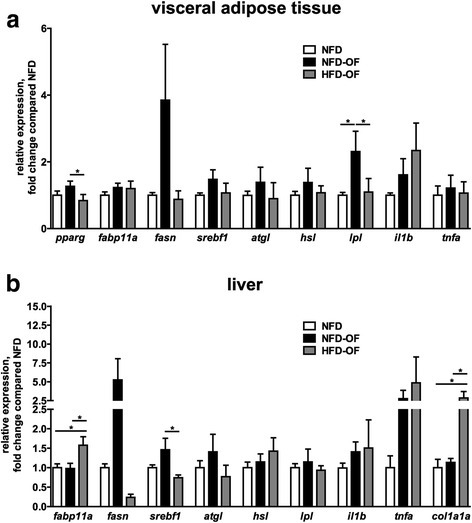
Diet-induced obesity is accompanied by characteristic alterations in AT and liver marker genes. Expression of marker genes for lipid metabolism (*pparg*, *fabp11a*, *fasn*, *srebf1, atgl, hsl, lpl*), inflammation (*il1b*, *tnfa*) and fibrosis (*col1a1a*) was analyzed in visceral AT (**a**) and liver (**b**) after 8-week overfeeding of adult male zebrafish with NFD or HFD. Induction of obesity by NFD-OF and HFD-OF resulted in characteristic alterations in metabolic, inflammatory and fibrotic marker gene expression in visceral AT and liver of zebrafish. Data show results from 3 independent feeding experiments and are given as mean ± SEM. Statistical analyses was performed by One-Way ANOVA and Bonferroni post-test and significant *p*-values are indicated. *, *p* < 0.05; AT, adipose tissue; NFD, normal fat diet; HFD, high fat diet; OF, overfeeding

To assess the molecular differences between NFD-OF and HFD-OF fish in AT and liver in more detail, we determined the expression of marker genes for lipid storage and inflammation, which had been previously associated with different obesity phenotypes in rodents and humans [24]. Due to the small amount of subcutaneous AT especially in control zebrafish, we had to restrict the analyses of marker gene expression to visceral AT. In visceral AT, we did not detect significant alterations in the expression of the adipocyte differentiation marker *pparg* after overfeeding with NFD (NFD-OF) or HFD (HFD-OF). However, in HFD-OF fish *pparg* was significantly lower compared to NFD-OF fish. Furthermore, we analysed expression of the lipid storage markers *fabp11a*, which is the zebrafish ortholog of human *FABP4* [25–27], as well as *fasn* and *srebf1,* which is an upstream regulator of *fasn* expression. While *fabp11a* expression was only slightly increased in response to overfeeding, *fasn* and *srebf1* were upregulated in NFD-OF but not altered in HFD-OF fish. Similarly, expression of *lpl,* which is a lipoprotein lipase and a central regulator in lipid metabolism*,* was significantly higher in NFD-OF zebrafish but remained unchanged in HFD-OF zebrafish compared to the control group. In contrast, expression of the lipases *atgl* and *hsl* was not affected by NFD and HFD overfeeding. Analyses of the inflammatory markers *il1b* and *tnfa* revealed a tendential upregulation of *il1b* in HFD-OF fish indicating slightly increased AT inflammation, while *tnfa* remained unchanged (Fig. 4a). Analyses of the liver expression profile showed significantly increased *fabp11a* expression in HFD-OF but not NFD-OF zebrafish. Moreover, *fasn* expression in the liver was upregulated in NFD-OF but significantly downregulated in HFD-OF when compared to control zebrafish. In line with these data, *srebf1* was increased in NFD-OF and decreased in HFD-OF fish when compared with control fish. Expression of the lipases *atgl*, *hsl* and *lpl* and the inflammatory marker *il1b* was not changed in fish included in the NFD-OF or HFD-OF group. In contrast, *tnfa* expression in the liver was increased under both overfeeding conditions. In addition, *col1a1a* expression was increased only in response to HFD but not NFD overfeeding suggesting the presence of fibrosis in the liver of HFD overfed zebrafish (Fig. 4b).

## Discussion

We show here that overfeeding of adult zebrafish with either NFD or HFD results in an increase in body weight, which is at least partially due to an increased accumulation of body fat. Interestingly and importantly, the biological and metabolic phenotypes differ depending on the type of the diet. In contrast to NFD-overfed zebrafish, HFD-overfed zebrafish present adipocyte hypertrophy, especially in the visceral AT depot, ectopic lipid accumulation in the liver, and hyperglycaemia and crucial differences in the expression of marker genes for lipid metabolism, inflammation and fibrosis,– hence a phenotype commonly referred to as MUO.

Different models of diet-induced obesity in zebrafish provided evidence that the pathophysiological pathways underlying diet-induced obesity in zebrafish are comparable to that of mammals [13, 14]. Most of those studies compared lean and obese animals and were based on excessive overfeeding of adult zebrafish with their “natural” diet in aquaculture conditions, i.e. flakes or artemia. We have substantially extended the published protocols by subjecting zebrafish to specific diets, i.e. NFD or HFD, over an 8-week-period. Our results show that dependent on the type of diet, one can not only induce obesity but also specifically trigger the development of a MHO versus a MUO AT phenotype as indicated by crucial differences in numbers and sizes of subcutaneous and visceral adipocytes, blood glucose, triglyceride and cholesterol levels, liver lipid accumulation and transcript levels of lipid metabolism and inflammation markers.

Compared with the MHO phenotype, the MUO phenotype is associated with characteristic alterations in the metabolic and immune function in human AT. These alterations include decreased expression of genes involved in lipid metabolism and increased expression of markers of AT inflammation [28]. Similarly to human studies, we observed a significant downregulation in the AT expression of several marker genes involved in lipid metabolism including *pparg*, *fasn* and *lpl* in HFD-OF compared to NFD-OF zebrafish. However, based on the striking increase in *fasn* expression we observed in both AT and liver upon overfeeding of zebrafish with a normal fat diet one would have expected a similar increase in *srebf1* expression, which is an upstream transcription factor regulating *fasn* expression. However, *fasn* expression is not only regulated by Srebf1 but also by other upstream regulators, such as Usf [29]. Hence, we cannot exclude that these factors exert an additional impact on *fasn* expression. Nevertheless, the fact that we observed increased AT *lpl* expression in NFD-OF but not in HFD-OF fish further supports our model for the induction of metabolically distinct obesity phenotypes in zebrafish. Previous data suggested that *lpl* expression and activity in obese subjects are downregulated in an insulin-resistant compared to an insulin-sensitive state [30, 31], and that an increase of lipoprotein lipase in adipocytes improves glucose metabolism in HFD-induced obesity [32].

We observed an increase in plasma triglyceride and cholesterol levels and ectopic liver lipid accumulation in zebrafish overfed with HFD compared to zebrafish overfed with NFD. These results point to distinct metabolic phenotypes of the two groups. However, the difference in blood glucose levels between the groups was only modest and might reflect the short duration of the study. We assume that this modest increase in blood glucose might represent early obesity-related alterations in glucose metabolism but not a manifestation of the disease. In fact, this assumption is underlined by the results from immunoblot analyses showing enhanced Thr308 phosphorylation of Akt (Thr307 for the zebrafish protein) in the HFD-OF group. Unfortunately, we cannot provide data on Ser473 phosphorylation of Akt since we were lacking an antibody specifically detecting the zebrafish protein. Furthermore, it would be interesting to analyse how Akt phosphorylation in liver tissue of zebrafish subjected to NFD and HFD overfeeding is affected by an insulin challenge. Both, enhanced Thr308 and Ser473 phosphorylation of Akt have been associated with an early state of insulin resistance upon HFD overfeeding [33–36]. In our opinion, the observation of increased Thr308 phosphorylation of Akt in liver tissue upon HFD overfeeding might represent a valuable strength of the study protocol allowing the analyses of obesity-related alterations in AT and liver which occur with AT accumulation before or during the development of obesity-related metabolic alterations. Hence, the model described in this study provides a unique tool to study physiological mechanisms involved in maintaining or disrupting metabolic health in obesity. The better understanding of these mechanisms might enhance the identification of novel therapeutic approaches specifically targeting the metabolic phenotype of obese patients. In this context, the zebrafish might be a particularly well-suited model organism. They share a considerable amount of genetic identity with humans and sophisticated mutagenesis, transgenesis, and screening strategies are available and can be used with an economy that is not possible in other vertebrates [10].

It should be noted that for the establishment of the short-term feeding protocol described in this study, we used male zebrafish only. Previous short-term and long-term analyses suggested that female zebrafish show a similar response to diet-induced obesity compared to male zebrafish with increased body length, body weight and BMI although to different extent [13, 14]. Reason for this might be that female zebrafish constantly produce eggs, which contain large amounts of lipids. In fact, it has been demonstrated that in adult female zebrafish ovaries can account for up to 29% of body weight compared to less than 2% for the testis in male zebrafish [13]. Because of this measurement of weight and body fat content in female adult zebrafish does not necessarily reflect AT accumulation but may be an indicator of oocyte growth as well. Since all zebrafish included in this study were either histologically analysed or subjected to organ preparation for expression analyses we can exclude that the occurrence of diet-induced sex reversal as a potential bias. It might be of interest for future studies to analyse whether the here provided feeding protocol is informative for both sexes. In this regard, the analysis of never-mated female zebrafish might provide important information on the transferability of the method to female zebrafish. Furthermore, obesity development and progression might be influenced by the age of fish, the genetic background and the body weight state at the start of the experiment. The here described protocol was established using 3 to 6 months old zebrafish of the AB strain. For the use of a different zebrafish strain, such as Tu, TL or WIK, specific parameters such as amount of food or duration of feeding experiment may need to be adjusted. Given the simplicity of the feeding protocol and the detailed phenotypic characterization described in our study, we believe that it can be easily applied to answer the questions whether sex, age or genetic background of zebrafish influence the response to NFD and HFD overfeeding and the associated development of metabolically distinct obesity phenotypes.

Our study is strengthened by the extensive phenotypic characterization including determination of total body fat content, adipocyte numbers and sizes in both subcutaneous and visceral AT, blood glucose, triglyceride and cholesterol levels, ectopic lipid accumulation in the liver and expression levels of marker genes for lipid metabolism and inflammation, which allows the identification of diet-dependent metabolically distinct obesity phenotypes.

## Conclusions

In summary, we have established a protocol for the induction of MHO versus MUO in zebrafish providing an attractive model to study regulatory mechanisms underlying the determination of distinct obesity-related AT phenotypes and metabolic state.
